# Dental Items

**Published:** 1871-04

**Authors:** 


### EDITORIAL DEPARTMENT.

---

*Dental Items.*—At a recent meeting of the Medical Society, of London, Mr. Napier read a paper on "An Improved Method of Filling Teeth," and exhibited some specimens in which cavities caused by decay were severally filled up with hippotamus ivory, mother of pearl, and India rubber, vulcanized to the consistence of ebony. Mr. Napier desires to obviate the necessity for using metal in any form for filling teeth, and read this paper with a view to prove the importance of the object he advocated. He argues that one of the principle causes of chronic inflammation in teeth that have been filled according to the method now in general use, is that metal is a readier conductor of heat and cold than the natural substance of which a tooth is composed. The improvement he advocated would benefit both operator and patient. We are of the opinion that time will prove this so-called improvement to be no better than many others which have heretofore been brought to the notice of the profession as substitutes for metal.

The "*Canada Journal of Dental Science*" publishes an article on the "Inflammability of 'the new base 'Pyroxyline' " from Dr. J. Neelands, in which he says:

"It may not be very generally known among dentists what an extremely inflammable substance pyroxyline is, and how easily a set of teeth may be destroyed by coming in contact with anything that is ignited. A short time ago an acquaintance of mine came up to the office in the evening, and I took him into the laboratory and showed the new kind of base I was using for artificial teeth. We each sat down and smoked a cigar. Just as we had entered the hall, about to leave the office, on turning round we observed the room we had just left brilliantly illuminated almost equal to that produced by the magnesian light. I rushed back and found my pyroxyline plate in a blaze and being rapidly consumed. On making an examination I discovered that when we left the room I happened to lay a lighted cigar in close proximity to the case of teeth, which set it on fire. It will require some degree of care on the part of those using this material not to allow lighted cigars, matches, or anything of that sort, to come in contact with it when out of the mouth. It might prove somewhat hazardous for an individual wearing a set of teeth made of this material to accidentally put *the wrong end* of a lighted cigar in his mouth.

Dr. W. H. Eames in the "*Missouri Dental Journal*," gives Hale & Eames' "Method of Casting Aluminum Plates," which any member of the profession is at liberty to use in his practice, free of charge. It is as follows:

"The impression is taken in the ordinary manner, with plaster or wax. If an air-chamber be desired, the form of it is cut in the impression. The model is made of plaster and French chalk, equal parts; fine white sand may be used instead of the chalk, but it is not so good. The model should be about one and one-half inches thick, and of such form as to draw readily from a plaster mould formed around it. While the model is yet soft, a small eyelet, made from ordinary wire, should be set in the centre of the back, opposite to the face. It should be completely buried in the substance of the model, so that it will be no more than flush with the surface when the model is trimmed. In trimming, cut away from around the eyelet a little, so that you can grasp it with a pair of pliers; the object of this eyelet being to aid in the removal of the model from the flask. Grind the teeth and articulate them as for rubber; place them in position with the trial-plate on the model. The trial or model plate should be made of sheet tin, lead, or wax. If wax be used, it should not be thicker than No. 20 gold plate. There should be no surplus wax, except at a point just back of the tooth or teeth. The trial-plate may pass up underneath the gum of a single tooth, but in no case should a particle of wax overlap the gum or edges of the teeth. When the case is ready for the flask, the plaster teeth, if there be any on the model, should be trimmed off, and if there be any under-cuts that would interfere with the removal of the model from the mold in the flasks, they should be filled up, or better, cut out. The sides and other exposed surfaces of the model are now to be covered with tin foil, to prevent the plaster adhering to it in flasking. Varnish should never be used for this purpose in this process. The foil is made to adhere by moistening the surface with saliva. It should not cover any portion of the artificial teeth. The flask consists of an ordinary cast-iron sand–ring, about two or two-and-a-half inches in depth, of the form of the letter D, one end larger than the other. They may be had at any of the dental depots in nests of four sizes. A bottom is formed, of Russia sheet iron, to fit into the small end, as follows: A piece is cut the shape of the end to be fitted, but half an inch larger. Slit the edge all round to the depth of half an inch and turn it up at right angles with the surface; this turned-up edge passes up into the flask and retains the bottom when the case is flasked. A hole one inch in diameter, of the form of the bottom, should be cut from the centre. To flask the case, place the model, face upwards on a marble slab or piece of glass; several pieces of fine wire, about two inches in length, should now be set in the surface of the tried plate at right angles with it. If the case be one of two or more teeth, one tooth in a place, a wire shonld be set into the wax immediately back of each tooth. One wire will be sufficient for a block of three teeth; two or three wires should be distributed over the surface of the plate. The object of the wires is to form outlets for the escape of the oxygen which is forced out of the metal, when the flask is closed under pressure. Place the ring or flask small end upwards around the model. Mix an investment of equal parts of plaster and French chalk, or white sand, to about the consistency of thick cream; fill round the model in the ring carefully, allowing no air-bubbles to be retained. When the flask is full, place the bottom in position, letting the wires pass through the hole in the centre; be particular to have the bottom flush with the edge of the flask. When the investment is hard, remove the wires by drawing them out, warm the case slightly, (not enough to melt the wax), grasp the eyelet in the model with a pair of pliers, and tap the edge of the ring gently until the model is loosened, it can then readily be withdrawn. Remove the trial-plate and every particle of wax and tin from the mold and the model; cut small outlets in the sides of the model for surplus metal; with a small unannealed broach make three or four vents near the centre of the model by passing the broach through from the back to the surface. The model and mould should now be thoroughly dried; this may be done by placing them separately over a gas-stove or in an oven. When dry, put them together, place the case in a charcoal fire, heat up gradually to red heat. Having previously prepared the metal by casting it into an ingot, in size sufficient for the case, melt it in an ordinary crucible; when melted, and the case is red hot, take the case from the fire, remove the model from the mould by means of the eyelet and a pair of long nosed pliers, blow out the ashes and dust that may have fallen into the mould, and pour in the metal, return the model, place the case in an ordinary rubber press and force the model down slowly, till flush with the top of the ring; let it stand till cold. The case can now be taken from the flask and finished. To do this, first saw off all the surplus metal round the edges; dress down the surface with burs and scrapers, and finish the same as rubber, except the polishing, which is best done with the burnisher."

Dr. C. J. Essig in the "*Dental Cosmos*" gives the following method of "Obtaining Impressions of Difficult Partial Cases," as follows:

"To obtain correct impressions of difficult partial cases, where the position of the remaining natural teeth form dovetailed or keystone shaped spaces, or where by the recession of the gums, we find teeth with narrow necks and broad, cutting edges, a form of tooth which cannot but increase the difficulty of securing an accurate model of the mouth, the use of plaster will be found to produce the best results, both as to correctness and comfort to the patient. In many of these cases, especially in the mouth of aged persons, some of the remaining teeth may be found to be loose from the recession of the gums, and the surrounding tissue so inflamed and sensitive, that even the pressure necessary to obtain an impression in wax would cause pain to the patient and danger to the loose teeth.

"An impression cup should first be selected of the proper size and shape,—those with the flat floor are best for partial cases; the plaster should be mixed thin, almost as thin as water, adding chloride of soda to facilitate setting. Plaster mixed in this manner does not become as hard and unyielding as that mixed merely to saturation. Now oil the cup so that so that it will readily separate from the impression when hard, fill the cup as soon as the plaster thickens sufficiently, then with a small spatula, place a layer of the soft plaster in upon the palatine surface, otherwise by inclosing the air in the deep portion of the arch the accuracy of the impression may be impaired. After this precaution, the cup is placed in the mouth, and gently pressed up until its floor comes into contact with the teeth.

When the plaster is sufficiently hardened, remove the cup, which from its having been oiled, is done without difficulty; with the thumb and index finger break off the outside walls, the portion covering the palatine surface is then removed by the use of a a blunt, steel spatula, curved at the end in the form of a hook. The pieces are then placed back into the cup, where they will be found to articulate with perfect accuracy.

"Should the first attempt be rendered futile, by a tendency to nausea, or troublesome gagging on the part of the patient, camphor-water, used as a gargle, will, in nearly every case, prove an effectual remedy. (The credit of this valuable discovery is due to Dr. Louis Jack.") (We are of the opinion that the credit belongs more properly to Prof. P. H. Austen, whose method of taking impressions of partial cases has been demonstrated in the Baltimore College for years. Ed. Journal.)

"In cases of fracture of the inferior maxillary, it is often necessary to obtain an accurate impression, in order that an apparatus may be constructed which, by clasping the teeth, will assist in holding the portions together until nature brings about a sufficient union. In such cases we could not think of forcing a mass of wax into the mouth and down around the teeth; indeed, the very pressure necessary to do so would force apart the broken bones, especially if the fracture be a compound one. By far the better plan would be to employ plaster, in the manner described for difficult partial cases.

